# Polyphenols in Litchi (*Litchi chinensis* Sonn.) Seeds: Comparison of Profiles Among Five Cultivars and Evaluation of Anti‐Inflammatory, Analgesic Potentials

**DOI:** 10.1002/fsn3.72200

**Published:** 2026-07-31

**Authors:** Fanke Zeng, Yuxiang Zhu, Xiaoyan Li, Ruyi Li, Lei Pan, Yaping Dai, Shaodan Peng, Lei Fang, Jihua Li, Wei Zhou

**Affiliations:** ^1^ Key Laboratory of Tropical Crop Products Processing of Ministry of Agriculture and Rural Affairs, Agricultural Products Processing Research Institute Chinese Academy of Tropical Agricultural Sciences Zhanjiang Guangdong China; ^2^ College of Food Science Fujian Agriculture and Forestry University Fuzhou Fujian China

**Keywords:** analgesic, anti‐inflammatory, A‐type procyanidin dimers, cultivar differentiation, litchi seeds, phloridzin

## Abstract

Litchi seeds are by‐products produced from the fruit consumption, which contain rich phenolic compounds and have been traditionally used in inflammatory pain relief. This study sought to reveal phenolic profiles in the litchi seeds from different cultivars and explore their contributions to anti‐inflammatory, analgesic potentials. Purified polyphenols were derived from five cultivars of litchi seeds, including Baitangying (BTY), Baila (BL), Feizixiao (FZX), Heiye (HY), and Huaizhi (HZ). Fifteen flavonoids, two phenolic acids, one coumarin, and two tannins were characterized using UPLC‐Q/TOF‐MS, while procyanidin A_1_, procyanidin A_2_, and phlorizin were identified as major phenolic compounds in HPLC. HY contained relatively higher phenolic contents. The polyphenols from different cultivars effectively inhibited LPS‐induced generation of ROS, NO, IL‐6, TNF‐*α*, and PGE_2_ in RAW 264.7 macrophages, which was tightly associated with the contents of procyanidin A_1_, procyanidin A_2_, and phlorizin. Procyanidin A_1_ played a relatively significant role, which could strongly bind to iNOS, COX‐2 and their mixture with procyanidin A_2_ and phlorizin exhibited better effect in the NO inhibition. Furthermore, the litchi seed polyphenols were demonstrated to inhibit xylene‐induced ear edema and acetic acid‐induced abdominal writhing in mice. Thus, the litchi seed polyphenols possessed potentials to be utilized as anti‐inflammatory and analgesic agents.

## Graphical abstract

Among the purified polyphenols derived from five cultivars of litchi seeds, the Heiye cultivar contained relatively higher phenolic contents. The litchi seed polyphenols exhibited anti‐inflammatory and analgesic potentials in vitro and in vivo, which were tightly associated with the major compounds including procyanidin A_1_, procyanidin A_2_, and phlorizin.

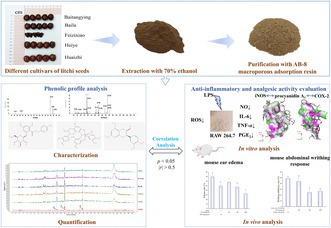

**Abbreviations**
BL: Baila litchi seed polyphenols
BTY: Baitangying litchi seed polyphenols
CCK‐8: cell counting kit‐8
COX‐1: cyclooxygenase‐1
COX‐2: cyclooxygenase‐2
CVB3: coxsackie virus B3
DCFH‐DA: 2,7‐dichlorodihydrofluorescein diacetate
DEX: dexamethasone
DMEM: Dulbecco's Modified Eagle Medium
ECE: epicatechin equivalents
ESI: electrospray ionization
FBS: fetal bovine serum
FZX: Feizixiao litchi seed polyphenols
GAE: gallic acid equivalents
HPLC: high performance liquid chromatography
HRF: heterocyclic ring fission
HSV‐1: herpes simplex virus 1
HY: Heiye litchi seed polyphenols
HZ: Huaizhi litchi seed polyphenols
IACUC: Institutional Animal Care and Use Committee
IDA: information‐dependent acquisition
IL‐6: interleukin‐6
iNOS: inducible nitric oxide synthase
LPS: lipopolysaccharide
MAPK: mitogen‐activated protein kinases
NF‐kB: nuclear factor kappa B
NO: nitric oxide
Nrf2: nuclear factor erythroid 2‐related factor 2
PBS: phosphate buffered saline
PGE_2_
: prostaglandin E_2_
QM: quinone methide
RDA: Retro‐Diels‐Alder
RE: rutin equivalents
RMSD: root mean square deviation
ROS: reactive oxygen species
TFC: total flavonoid content
TNF‐*α*
: tumor necrosis factor‐alpha
TPAC: total proanthocyanidin content
TPC: total phenolic content
UPLC‐Q/TOF‐MS: ultra‐high performance liquid chromatography‐quadrupole time‐of‐flight mass spectrometry

## 1 Introduction

Litchi ( *Litchi chinensis* Sonn.) is a subtropical and tropical plant of the Sapindaceae family. The seeds of litchi mainly produce from the consumption of litchi fruits and have been widely used in traditional medicine of China and India to alleviate inflammation and pain associated conditions, including orchitis, swelling, neuralgia, and abdominal pain during menstruation (Chukwuma et al. 2021; Yang et al. 2011). They contain various bioactive ingredients, including flavonoids, terpenoids, phenolics, saponins, and oils, which are related to the physiological activities (Xiang et al. 2022). However, there are more than 30 commercial cultivars of litchi throughout the world, and the differences concerning components and activities of the seeds remain indistinct (Jiang et al. 2021).

Polyphenols are naturally secondary metabolites found in plant sources, which have garnered increasing attention for their potential benefits to human health (Mitra et al. 2022). Litchi seeds represent a rich source of phenolic compounds. Polyphenol‐rich extract from litchi seeds could reduce systolic blood pressure and improve hypertension‐induced renal damage by suppressing inflammation and oxidative stress (Yao et al. 2021). Total flavonoids of litchi seeds could inhibit breast cancer metastasis (Yang et al. 2025). Ren et al. (2011) found that the flavanone compounds from litchi seeds showed activity in *α*‐glucosidase inhibition. A‐type proanthocyanidins from litchi seeds exhibited in vitro antioxidant and antiviral activity against coxsackie virus B3 (CVB3) and herpes simplex virus 1 (HSV‐1) (Xu et al. 2010). In the inflammatory condition induced by microorganisms or tissue damage, immune cells such as macrophages release reactive oxygen species (ROS), nitroxidative stress, and pro‐inflammatory cytokines. The polyphenols reduce inflammation mainly through acting as antioxidants, interfering with oxidative stress signaling, and suppressing pro‐inflammatory signaling transductions (Zhang and Tsao 2016). Pathological pain can be initiated after inflammation. For instance, prostaglandin (PG) E_2_ synthesis is mediated by cyclooxygenase‐1 (COX‐1) or COX‐2 upon activation of transcription factors such as nuclear factor kappa B (NF‐kB) and mitogen‐activated protein kinases (MAPK). During inflammation, increased production of PGE_2_ contributes to neuronal sensitization leading to pain (Ilari et al. 2022). Although pain relief is one of the most important medicinal applications of litchi seeds, the molecular basis of phenolic compounds to exhibit anti‐inflammatory and analgesic potentials remains unknown.

In this study, the seeds of five commercial litchi cultivars were collected. Polyphenols were extracted and purified from the litchi seeds. Ultra‐high performance liquid chromatography‐quadrupole time‐of‐flight mass spectrometry (UPLC‐Q/TOF‐MS) was used to characterize the phenolic compositions, and high performance liquid chromatography (HPLC) was applied to quantify the contents of major phenolic compounds. Anti‐inflammatory, analgesic potentials, and molecular contribution of the polyphenols in litchi seeds were evaluated in RAW 264.7 macrophages and mice. The results of this study will provide a scientific explanation for the traditional efficacy of litchi seeds and suggest the potential application of their polyphenols.

## 2 Materials and Methods

### 2.1 Materials and Reagents

Five cultivars of fresh litchi fruits (Baitangying, Baila, Feizixiao, Heiye, Huaizhi) were identified and collected at the National Field Genebank for Litchi (Maoming, China, 111.00° E, 21.78° N) in June 2024. The voucher herbarium specimens of five cultivars have been numbered as 01391148, 01581972, 01391141, 01391139, and 01391128 respectively and preserved in Herbarium, Institute of Botany, CAS. Dexamethasone and Folin–Ciocalteu reagent were supplied by Solarbio Science & Technology Co. Ltd. (Beijing, China). Aladdin (Shanghai, China) provided acetic acid, xylene, aspirin, and acetonitrile, methanol, formic acid of HPLC quality. Fetal bovine serum (FBS), Dulbecco's Modified Eagle Medium (DMEM), lipopolysaccharide (LPS), penicillin–streptomycin, and phosphate buffered saline (PBS) were obtained from Gibco (New York, USA). Cell counting kit‐8 (CCK‐8), Griess assay kits, and 2,7‐dichlorodihydrofluorescein diacetate (DCFH‐DA) were purchased from Beyotime Biotechnology (Shanghai, China). Murine interleukin‐6 (IL‐6) and tumor necrosis factor‐alpha (TNF‐*α*) ELISA kits were supplied by NeoBioscience Technology Co. Ltd. (Shenzhen, China). Elabscience Biotechnology Co. Ltd. (Wuhan, China) offered ELISA kits of murine PGE_2_. AB‐8 macroporous adsorption resin, gallic acid, rutin, epicatechin, procyanidin A_1_, procyanidin A_2_, and phlorizin were supplied by Shanghai Yuanye Bio‐Technology Co. Ltd. (China). All other chemical reagents used were of analytical grade. Deionized water was prepared using a Q‐POD purification system (Millipore, France).

### 2.2 Extraction and Purification of Polyphenols

**FIGURE 1:**
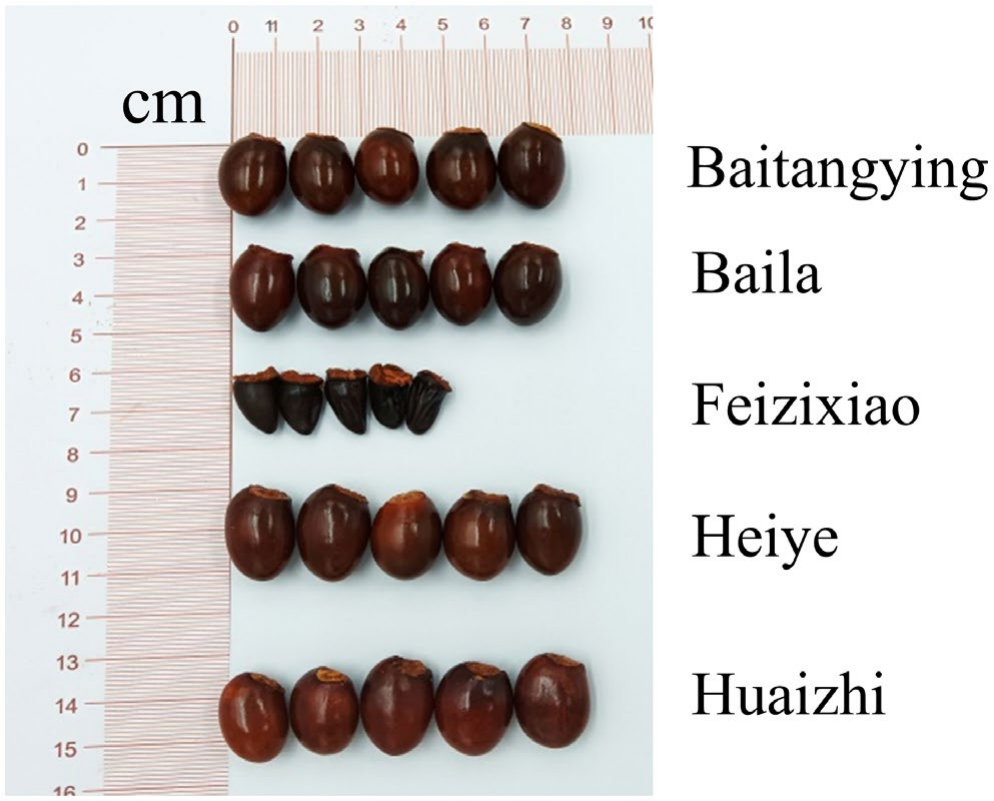
Morphology of seeds from five representative litchi cultivars: ‘Baitangying’, ‘Baila’, ‘Feizixiao’, ‘Heiye’, and ‘Huaizhi’.

Litchi seeds were manually separated from the fruits (Figure 1), dried, and finally ground into powder. The samples of litchi seed powder (100 g) of different varieties were subjected to extraction using 1000 mL of 70% ethanol (v/v) at 70°C for 1 h. After rotary evaporation and freeze‐drying of the supernatant, the crude polyphenol extracts were obtained. Subsequently, the crude extracts were purified using an AB‐8 macroporous adsorption resin. Sequential elution was performed with purified water and 20% ethanol (v/v) to remove water‐soluble impurities and polysaccharides. Finally, the 70% ethanol‐eluted fraction was collected, concentrated by rotary evaporation, and freeze‐dried to obtain the purified polyphenols. The purified polyphenols derived from the seeds of Baitangying, Baila, Feizixiao, Heiye, and Huaizhi litchi varieties were designated as BTY, BL, FZX, HY, and HZ, respectively. The yields of BTY, BL, FZX, HY, and HZ were 0.30%, 0.59%, 0.40%, 0.45%, and 0.26%, respectively.

### 2.3 Determination of Total Phenolic Content (TPC)

Determination of TPC of each sample was carried out according to the method of Laily et al. (2015). 0.1 mL of BTY, BL, FZX, HY, or HZ solution was combined with 2.9 mL of distilled water and 0.5 mL of diluted Folin–Ciocalteu reagent. Five minutes later, 0.75 mL of 20% Na_2_CO_3_ (m/v, g/mL) was added to the mixture. After stirring, the liquid was left at room temperature in dark for 30 min. Finally, the absorbance was measured at 765 nm with a UV/VIS spectrophotometer (MAPADA‐M8, China). The total phenolics were calculated by using the gallic acid calibration curve (0–300 μg/mL) and expressed as milligram gallic acid equivalents of per gram sample in dry weight (mg GAE/g DW).

### 2.4 Determination of Total Flavonoid Content (TFC)

TFC was determined according to the procedure described by Zheng et al. (2022). 0.5 mL of BTY, BL, FZX, HY, or HZ solution was mixed with 0.2 mL of 5% NaNO_2_. After 6 min, the sample was incubated with 0.2 mL of 10% AlCl_3_ for another 6 min. The mixture was then vortexed with 0.5 mL of 4% NaOH and 5 mL of distilled water. Then the sample was kept for 15 min at room temperature. Absorbance of the liquid was measured at 510 nm. A calibration curve was graphed using rutin (50–500 μg/mL) and the results were expressed as milligram rutin equivalents of per gram sample in dry weight (mg RE/g DW).

### 2.5 Determination of Total Proanthocyanidin Content (TPAC)

The assay was performed according to the method described by Kessy et al. (2016). One milliliter of BTY, BL, FZX, HY, or HZ solution was mixed with 2.5 mL of 0.5% vanillin‐methanol (v/v) solution and 20% H_2_SO_4_‐methanol (v/v) solution for 20 min in a 30°C water bath. To determine the proanthocyanidin content, the sample was analyzed by spectrophotometry, with absorbance recorded at 500 nm. A standard curve was prepared using epicatechin (200–600 μg/mL), and the results were expressed as milligram epicatechin equivalents per gram sample in dry weight (mg ECE/g DW).

### 2.6 UPLC‐Q/TOF‐MS Analysis

BTY, BL, FZX, HY, or HZ was prepared as phenolic solutions at a concentration of 0.7 mg/mL for subsequent UPLC‐Q/TOF‐MS (AB Sciex Triple TOF 5600+) analysis. Quadrupole mass axis calibration was conducted by injecting calibration solution and matching the calibrated ions. The error was generally controlled within 0.2 u. The mobile phase was composed of 0.05% formic acid in water (Eluent A) and acetonitrile (Eluent B), with a gradient elution performed at a flow rate of 0.3 mL/min: 0.01–4 min, 5%–10% B; 4–20 min, 10%–20% B; 20–40 min, 20%–30% B; 40–55 min, 30%–50% B; 55–56 min, 50%–95% B; 56–60 min, 95%–100% B; 60–60.01 min, 100%–5% B; a 10‐min re‐equilibration. A 2 μL aliquot of each sample was loaded onto the column (Waters ACQUITY BEH C18, 150 mm × 2.1 mm, 1.7 μm; 35°C) for chromatographic separation. Electrospray ionization (ESI) source was operated in negative ion mode at 550°C, with additional MS parameters set as described below: Information‐dependent acquisition (IDA) mode; mass spectral range, *m*/*z* 50–1500; capillary energy, −35 V; source voltage, −4.5 kV. Precursor masses and MS/MS peaks were compared with those of components in 5,064,224 Natural Products HR‐MSMS Spectral Library 1.1.

### 2.7 Quantification by HPLC

Quantification mainly referred to Cheng's research (2023) with slight modifications in the elution procedure. Briefly, HPLC (Shimadzu LC‐20AT, Japan) equipped with a column oven (CTO‐10AS) and a photodiode array detector (SPD‐M20A) was employed to quantify the principal phenolic compounds in BTY, BL, FZX, HY, and HZ. The separation was performed using a Zorbax SB‐C18 column (4.6 × 250 mm, 5 μm, Agilent, USA). The mobile phase A and B consisted of 1% formic acid in water and acetonitrile, respectively. The gradient condition was 10%–15% B in 0.01–5 min, 15%–20% B in 5–20 min, 20%–30% B in 20–45 min, 30%–60% B in 45–50 min, 60%–90% B in 50–55 min, 90%–10% B in 55–58 min, and a 6‐min re‐equilibration. The sample volume was 10 μL, with a flow‐rate set at 0.8 mL/min. For the preparation of the calibration curve, the standard stock solution was prepared in methanol containing procyanidin A_1_ (0.16–4.22 μg, *y* = 1,427,184.78*x* + 100,344.56, *R* ^2^ = 0.9987), procyanidin A_2_ (1.06–5.20 μg, *y* = 962,276.62*x*—24,409.51, *R* ^2^ = 0.9980), and phlorizin (0.17–1.95 μg, *y* = 4,748,241.66*x* + 222,924.61, *R* ^2^ = 0.9998) and the UV detector was set at 280 nm. Acceptable signal to noise ratios were set as 3:1 for the estimation of the LODs and 10:1 for the LOQs (Giusti et al. 2017). The LODs of the adopted method ranged from 0.0004 μg for phloridzin to 0.005 μg for procyanidin A_2_. The LOQs ranged from 0.001 μg for phloridzin to 0.018 μg for procyanidin A_2_. In the samples of BTY, BL, FZX, HY, and HZ, run‐to‐run precision %RSDs ranged from 0.73% to 2.83% for procyanidin A_1_, ranged from 0.71% to 1.95% for procyanidin A_2_, and ranged from 0.04% to 8.60% for phloridzin. Individual compounds were quantified in mg/g DW through comparison with the calibration curve.

### 2.8 Evaluation of Anti‐Inflammatory and Analgesic Potentials in Vitro

#### 2.8.1 Cells and Culture Conditions

The RAW 264.7 murine macrophage line was obtained from the Guangdong Medical University (Zhanjiang, China) and cultured at 37°C in DMEM containing 10% FBS and 1% penicillin–streptomycin under a humidified atmosphere of 5% CO_2_.

#### 2.8.2 Cell Viability Assay

The CCK‐8 assay was carried out to estimate the cytotoxicity of BTY, BL, FZX, HY, HZ, and individual phenolic compounds against the RAW 264.7 macrophages. After inoculation in a 96‐well microplate at a density of 10^5^ cells/mL, the macrophages were intervened with 100 μL of BTY, BL, FZX, HY, HZ (1, 5, 10 μg/mL), or individual phenolic compounds (5 μg/mL) for 24 h. Then, 100 μL of CCK‐8 solution was added and incubation was resumed for 1 h. The cell viability, expressed as absorbance percentage compared against the untreated control macrophages, was determined at 450 nm by a microplate reader (BioTek, Synergy H1, USA).

#### 2.8.3 ROS Assay

The RAW 264.7 macrophages were inoculated at a density of 10^5^ cells/mL in a 6‐well plate, pretreated with BTY, BL, FZX, HY, or HZ (10 μg/mL) for 2 h, and stimulated with 1 μg/mL LPS for 24 h. Dexamethasone (DEX) was used as a positive control at 10 μg/mL. Then, they were washed with PBS and loaded with 10 μM DCFH‐DA detection reagent. After incubation in darkness for another 20 min at 37°C, the cells were washed three times with PBS. The images for the cells were visualized using a fluorescence microscope (Leica, DMI6000 B, Germany) at excitation and emission wavelengths of 488 and 525 nm, respectively. The fluorescence intensity representing intracellular ROS levels was analyzed by Image J software.

#### 2.8.4 Nitric Oxide (NO) and Pro‐Inflammatory Cytokines (IL‐6, TNF‐α, and PGE_2_) Measurement

The RAW 264.7 macrophages at a density of 10^5^ cells/mL were cultured in 96‐well plates for 24 h. Different concentrations of BTY, BL, FZX, HY, HZ (1, 5, 10 μg/mL), or individual phenolic compounds (5 μg/mL) were prepared to give a total volume of 100 μL in each well. Following a 2‐h pretreatment period, the cells were exposed to 1 μg/mL LPS for 24 h. DEX was used as a positive control at 10 μg/mL. Cell‐cultured supernatants were collected for the quantification of NO and pro‐inflammatory cytokines, including IL‐6, TNF‐*α*, and PGE_2_. Protocols supplied with Griess and ELISA assay kits were referenced for the assay procedure.

#### 2.8.5 Molecular Docking Simulation

The SDF file of procyanidin A_1_ was obtained from the PubChem database. The structures of *Mus musculus* target proteins iNOS (inducible nitric oxide synthase, PDB ID: 2ORO; resolution: 2.00 Å) and COX‐2 (PDB ID: 5W58; resolution: 2.27 Å) were retrieved from the PDB database. The target proteins were optimized using PyMOL‐2.1.0 by removing water molecules and small molecule ligands, followed by hydrogen and charge addition with AutoDock Tools‐1.5.6, and saved in PDBQT format. The value of exhaustiveness was set to 8. A grid box completely covering the entire iNOS or COX‐2 protein surface was constructed, and the central coordinates were *x* = 65.635 Å, *y* = −14.402 Å, *z* = 49.875 Å and *x* = −3.345 Å, *y* = −23.515 Å, *z* = −73.016 Å, respectively. Using the target proteins as receptors and procyanidin A_1_ as the ligand, molecular docking was performed with Vina‐2.0 integrated in PyRx software to calculate binding energies. The results were visualized using PyMOL. To ensure the reliability of the molecular docking protocol, a re‐docking validation was also performed. The co‐crystallized ligands of iNOS and COX‐2 were re‐docked into their respective active sites using the same docking settings described above. The resulting poses were compared to the crystallographic ligand conformations by calculating the root mean square deviation (RMSD) values. The docking was considered valid when the RMSD was below 2.0 Å (Shoaib et al. 2023). When the binding energies were lower than −7.0 kcal/mol, the receptor and ligand have relatively strong and stable binding affinity (Deng et al. 2024).

### 2.9 Evaluation of Anti‐Inflammatory and Analgesic Potentials in Vivo

#### 2.9.1 Animals and Feeding Conditions

Eight‐week‐old male and female Kunming mice weighing 20 ± 2 g were purchased from the SPF (Beijing) Biotechnology Co. Ltd. (China). The mice were acclimated for 1 week and deprived of diet for 12 h prior to any trial. They were maintained under controlled temperature (25°C ± 2°C) and humidity (55%–60%) in a 12‐h light/dark cycle, with free access to water and regular diet. All the animal experiments complied with ARRIVE (Animal Research: Reporting of In Vivo Experiments) guidelines and protocol MDL2024‐12‐04‐03 was approved by the Institutional Animal Care and Use Committee (IACUC) of Kangtai Medical Testing Service Hebei Co. Ltd., which performed the animal experiments.

#### 2.9.2 Xylene‐Induced Mouse Ear Edema

The preventive effect of HY on acute inflammation in vivo was investigated in xylene‐induced mouse ear edema as described previously with slight modification (Zhao et al. 2018). Six mice (half male and half female) were allocated to each of the five experimental groups in this assay. Oryza Oil & Fat Chemical CO. Ltd. (2019) had investigated the acute toxicity of litchi seed extract, which was extracted with aqueous ethanol and guaranteed a minimum of 12.0% polyphenols. The median lethal dose (LD50) in mice was estimated to be more than 5000 mg/kg. A chemical substance with an LD50 within the range of 5000–15,000 mg/kg is considered to be practically nontoxic (Mukinda and Eagles 2010). Three groups were given HY orally at different doses [250, 500, and 1000 mg/(kg·day)], which might be effective doses according to previous research (Sindhu et al. 2021). One group orally received an anti‐inflammatory drug [DEX at 10 mg/(kg·day)], serving as a positive control. The remaining group served as a model control group, receiving orally physiological saline (0.9% NaCl, v/v). Each group's oral administration was conducted for 7 days continuously. One hour following the final administration, a volume of 20 μL of xylene was applied evenly to both the inner and outer surfaces of the right ear, with the left ear serving as a control. The mice were sacrificed 30 min after the xylene application, and both ears were excised. Circular ear tissue samples (5.0 mm in diameter) were punched out and weighed. The difference in weights between the right and left ear disks represented the development of edema.

Edema degree was calculated as follows:

$$\text{Edema rate}\;\left(\mathrm{ER},\%\right)=\left(W_{R}-W_{L}\right)/W_{L}\times 100$$

where *W*_*R*_ is the right ear disk weight, and *W*_*L*_ is the left ear disk weight of the same mouse.

Edema inhibitory activity was calculated according to the following formula:

$$\text{Edema inhibitory rate}\;\left(\%\right)=\left({\mathrm{ER}}_{\text{model control}}-{\mathrm{ER}}_{\text{treated}}\right)/{\mathrm{ER}}_{\text{model control}}\times 100$$

where ER is the edema rate for the model control or treated groups.

#### 2.9.3 Acetic Acid‐Induced Mouse Abdominal Writhing Response

To evaluate the effect of HY on the pain threshold, the writhes test in mice was carried out (Yu et al. 2019). The experimental design followed Section 2.9.2 and aspirin was used in the positive control. Following a 7‐day oral administration of the test solutions (physiological saline, HY, or aspirin), the mice received an intraperitoneal injection of 0.60% acetic acid at a dosage of 10 mL/kg. Three minutes after the injection, nociceptive behavior induced by acetic acid was quantified by recording the number of abdominal writhes within 10 min.

Pain inhibitory activity was expressed using the formula:

$$\text{Writhing inhibitory rate}\;\left(\%\right)=\left(N_{\text{model control}}-N_{\text{treated}}\right)/N_{\text{model control}}\times 100$$

where *N* is the number of writhes for the model control or treated groups.

### 2.10 Statistical Analysis

Results are expressed as mean ± standard deviation (SD) based on at least three independent replicates. Statistical analyses were conducted using SPSS 16.0. One‐way analysis of variance (ANOVA) followed by Duncan's multiple range test was applied at the confidence level *p* < 0.05. Pearson correlation analysis was used to assess the relationships between phenolic contents in BTY, BL, FZX, HY, HZ and their anti‐inflammatory, analgesic activities in RAW 264.7 cells.

## 3 Results and Discussion

### 3.1 TPC, TFC, and TPAC

Table 1 showed the results of TPC, TFC, and TPAC of purified phenolic extracts (BTY, BL, FZX, HY, or HZ) from various cultivars of litchi seeds. The TPC ranged from 599.64 ± 4.72 mg GAE/g DW in BL to 811.25 ± 13.89 mg GAE/g DW in HY. The lowest TFC of 250.00 ± 14.14 mg RE/g DW was monitored in HZ, in contrast to the highest values of 483.13 ± 23.63 mg RE/g DW in HY and 480.94 ± 1.33 mg RE/g DW in BL. The similar trend was observed in the proanthocyanidin assay. HY had the highest content of proanthocyanidins (849.95 ± 5.56 mg ECE/g DW), followed by FZX (755.56 ± 5.56 mg ECE/g DW) and BL (739.82 ± 11.12 mg ECE/g DW). Overall, HY exhibited significantly higher concentrations of phenols, flavonoids, and proanthocyanidins compared to the other cultivars (*p* < 0.05). In Yao's study (2021), the TPC and TFC in purified litchi seed extract were determined as 751.83 ± 34.32 mg GAE/g DW and 507.83 ± 8.56 mg RE/g DW, respectively. The TPC value aligned with the range observed in our study, whereas the TFC was notably higher. However, the cultivar information was limited. The TPAC in litchi had been mainly focused on the pericarp. For instance, Yang et al. (2022) reported a TPAC of 285.3 ± 9.8 mg ECE/g DW in litchi pericarp extract, which was markedly lower than that found in the seed extracts in our investigation. This suggested that litchi seeds might represent a more abundant source of proanthocyanidins.

**TABLE 1:** Total phenol, flavonoid, and proanthocyanidin contents in BTY, BL, FZX, HY, and HZ.

Variety	TPC (mg GAE/g DW)	TFC (mg RE/g DW)	TPAC (mg ECE/g DW)
HY	811.25 ± 13.89^a^	483.13 ± 23.63^a^	849.95 ± 5.56^a^
FZX	746.96 ± 3.79^b^	343.54 ± 11.19^b^	755.56 ± 5.56^b^
BL	599.64 ± 4.72^e^	480.94 ± 1.33^a^	739.82 ± 11.12^b^
BTY	691.61 ± 6.31^c^	349.58 ± 6.86^b^	633.63 ± 0.00^c^
HZ	656.79 ± 7.58^d^	250.00 ± 14.14^c^	574.63 ± 16.69^d^

*Note:* Mean ± SD (*n* = 3) labeled with different letters across the five varieties differ significantly (*p* < 0.05).

### 3.2 Phenolic Composition

UPLC‐Q/TOF‐MS has been widely employed for the identification and characterization of phenolic compounds. Twenty phenolic compounds (Library score > 80) present in BTY, BL, FZX, HY, and HZ samples were tentatively identified and characterized based on their precursor masses and fragmentation patterns compared with the database information and earlier published literature. These comprised flavonoids (15), phenolic acids (2), coumarins (1), and tannins (2), as detailed in Table 2.

**TABLE 2:** Characterization of phenolic compounds in BTY, BL, FZX, HY, and HZ by UPLC‐Q/TOF‐MS.

No.	Proposed compounds	Molecular formula	RT (min)	Molecular weight (u)	Precursor (*m/z*)	MS/MS product ions (*m/z*)	Library score
**Flavonoids**							
*Chalcones*							
1	Phloridzin	C_21_H_24_O_10_	18.976	436.14	435.13	273.15, 167.08	94.9
2	Naringin dihydrochalcone	C_27_H_34_O_14_	26.305	582.55	581.19	475.15, 273.08, 167.04, 123.04	94.6
3	Naringenin chalcone	C_15_H_12_O_5_	31.175	272.07	271.06	187.04, 151.01, 119.05, 107.01, 65.00	96.3
*Flavanones*							
4	Eriocitrin	C_27_H_32_O_15_	16.881	596.17	595.17	287.13, 151.04	93.2
5	Narirutin	C_27_H_32_O_14_	20.068	580.18	579.17	271.06	97.0
6	Neoeriocitrin	C_27_H_32_O_15_	21.805	596.53	595.17	287.06, 151.00	85.7
7	Naringin	C_27_H_32_O_14_	22.327	580.18	579.17	459.11, 313.07, 271.06	98.9
8	Naringenin	C_15_H_12_O_5_	31.166	272.07	271.06	177.02, 151.00, 119.05	97.6
*Flavonols*							
9	Quercetin‐*O*‐neohesperidoside	C_27_H_30_O_16_	16.294	610.52	609.15	301.03, 271.03	100.0
10	Rutin	C_27_H_30_O_16_	16.805	610.15	609.15	301.03, 271.03	99.4
11	Typhaneoside	C_34_H_42_O_20_	17.109	770.69	769.22	315.18, 299.02	100.0
12	Isoquercitrin	C_21_H_20_O_12_	17.501	464.10	463.09	301.03, 300.03, 271.03, 255.03	100.0
13	Quercitrin	C_21_H_20_O_11_	20.422	448.10	447.09	301.03, 300.03, 255.03, 151.00	99.3
*Flavanonols*							
14	Taxifolin	C_15_H_12_O_7_	16.006	304.06	303.05	177.04, 125.03	82.6
*Flavanols*							
15	Epicatechin	C_15_H_14_O_6_	7.571	290.08	289.07	245.08, 137.02	94.4
**Phenolic acids**							
*Hydroxybenzoic acids*							
16	Protocatechuic acid	C_7_H_6_O_4_	2.765	154.02	153.02	109.03, 108.02	94.2
*Hydroxycinnamic acids*							
17	Caffeic acid	C_9_H_8_O_4_	17.320	180.04	178.98	134.99	90.6
**Coumarins**							
18	Hydroxylated coumarin	C_9_H_6_O_3_	1.189	162.03	160.84	116.93	85.2
**Tannins**							
19	B‐type procyanidin dimer	C_30_H_26_O_12_	5.697	578.50	577.13	451.01, 425.09, 407.08, 289.08, 125.02	95.8
20	A‐type procyanidin dimer	C_30_H_24_O_12_	12.954	576.50	575.12	449.20, 423.18, 289.14, 285.11, 125.06	89.1

#### 3.2.1 Flavonoids and Tannins

Flavonoids constituted the most abundant class of phenolic compounds identified in the litchi seeds. Procyanidins were tentatively identified as predominant tannins in this study. Previous studies have shown that the primary flavonoids and tannins in the litchi seeds include phlorizin, rutin, epicatechin, procyanidins A_1_, and A_2_ (Kavya et al. 2025). The negative ion ESI‐MS spectrum of isolated Compound **1** exhibited a deprotonated molecular ion [M‐H]^−^ at *m*/*z* 435.13, which was consistent with a phloretin hexoside (phloridzin). A fragment ion observed at *m*/*z* 273.15 was attributed to the phloretin aglycon moiety (Hilt et al. 2003). Compound **9** and **10** eluted at 16.32 and 16.85 min, respectively. Both exhibited a deprotonated molecular ion [M‐H]^−^ at *m*/*z* 609.15. In MS/MS analysis, an intense fragment ion was observed at *m*/*z* 301.03 (quercetin aglycone), corresponding to the loss of a 308 u moiety. This mass shift was characteristic of rutinose or neohesperidose glycosidic units. Based on their elution order, Compound **9** was tentatively characterized as quercetin‐*O*‐neohesperidoside, and Compound **10** as quercetin‐3‐*O*‐rutinoside (rutin) (M'rabet et al. 2017). Compound **15** (epicatechin) displayed a [M‐H]^−^ ion at *m*/*z* 289.07. Fragment ions including *m*/*z* 245.08 [M‐H‐C_2_H_4_O]^−^ and *m*/*z* 137.02 [M‐H‐C_8_H_8_O_3_]^−^ were in agreement with the literature data (Maisto et al. 2022). As for procyanidins, A‐ and B‐type dimers could be distinguished by their parent ions [M‐H]^−^ at *m*/*z* 575 or 577, respectively (Appeldoorn et al. 2009). MS/MS analysis of Compound **20** (*m*/*z* 575.12) revealed characteristic fragment ions at *m*/*z* 125.06, 285.11, 289.14, 423.18, and 449.20, which were consistent with A‐type procyanidin dimers. The ions at *m*/*z* 125.06 and 449.20 originated from Heterocyclic Ring Fission (HRF). The ion at *m*/*z* 285.11 arose from quinone methide (QM) cleavage. The ion at *m*/*z* 423.18 was attributed to Retro‐Diels‐Alder (RDA) fragmentation pathway. MS/MS spectrum of Compound **19** (*m*/*z* 577.13) exhibited fragment ions at *m*/*z* 125.02, 289.08, 407.08, 425.09, and 451.01, which was indicative of B‐type procyanidin dimers. The signals at *m*/*z* 125.02 and 451.01 originated from HRF. Those at *m*/*z* 289.08 and 425.09 resulted from the cleavage of QM and RDA, respectively. Additionally, the ion at *m*/*z* 407.08 was generated by the loss of a water molecule from the fragment at *m*/*z* 425.09 (Li et al. 2025). Compound **11** showed a molecular ion peak [M‐H]^−^ at *m*/*z* 769.22. A fragment ion observed at *m*/*z* 315.18 corresponded to the aglycone isorhamnetin, formed by the loss of two rhamnose and one hexose moieties (total 454 u). Based on this fragmentation pattern, the compound was tentatively identified as isorhamnetin‐dirhamnopyranosyl‐hexoside (Typhaneoside) (El‐Zahar et al. 2022), which was originally identified in the litchi seeds. Typhaneoside has been an effective flavonoid for pain relief in Chinese medicines (Wang et al. 2023). Narirutin (Compound **5**) and naringin (Compound **7**) had also been detected in the litchi seeds in a previous study with parent ion [M‐H]^−^ at *m*/*z* 579.17 (Wang et al. 2011).

#### 3.2.2 Phenolic Acids

In our study, two types of phenolic acids were tentatively characterized, including hydroxybenzoic and hydroxycinnamic acids, which exist in fruits, vegetables, legumes, cereals, and their by‐products (da Silva et al. 2023). The hydroxybenzoic acids are characterized by a carboxyl group bonded directly to the benzene ring. Compound **16** was identified as protocatechuic acid, belonging to the class of hydroxybenzoic acid. Its secondary mass spectrum displayed a deprotonated molecular ion [M‐H]^−^ at *m*/*z* 153.02. Upon collisional activation, a secondary fragment was observed at *m*/*z* 109.03, resulting from the loss of a carbon dioxide (CO_2_) molecule from the carboxylic acid group. These results aligned with previously published data (Chen et al. 2012). Protocatechuic acid has been identified with moderate antioxidant activity in the litchi seeds by Wang et al. (2011). Compound **17** was identified as caffeic acid (a hydroxycinnamic acid), eluting at 17.26 min with a deprotonated molecular ion [M‐H]^−^ at *m*/*z* 178.98. The predominant fragment ion observed at *m*/*z* 134.99 resulted from decarboxylation ([M‐H‐COO]^−^), which was consistent with previous reports by Hossain et al. (2010). Caffeic acid has been widely characterized in the litchi pulp (Su et al. 2014; Zhang et al. 2013), however, the occurrence in the litchi seeds was rarely reported.

#### 3.2.3 Coumarins

Compound **18** was determined to be a hydroxylated coumarin, displaying a precursor [M‐H]^−^ at *m*/*z* 160.84. The relative abundance of the product ions of its various isomers is different. The major fragment obtained for Compound **18** was *m*/*z* 116.93 produced from loss of a CO_2_. Essentially no *m*/*z* 133 fragment was detected due to loss of a CO from the molecular ion. This characteristic was consistent with 5‐ and 7‐hydroxycoumarin (Weymarn and Murphy 2001), which were rarely reported in the litchi seeds.

### 3.3 Contents of Major Phenolic Compounds

**FIGURE 2:**
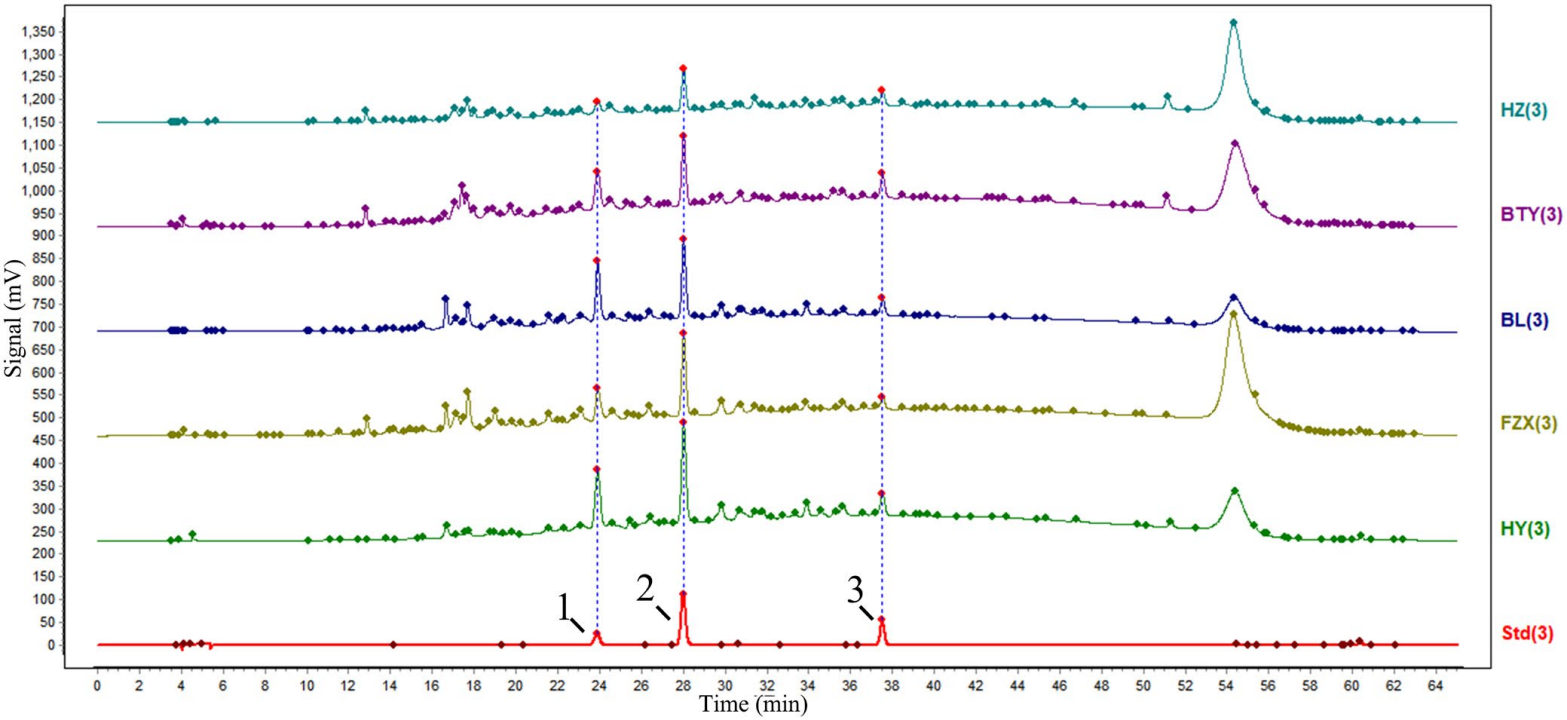
HPLC chromatograms of BTY, BL, FZX, HY, and HZ (Peak 1, 23.934 min, Procyanidin A_1_; Peak 2, 28.105 min, Procyanidin A_2_; Peak 3, 37.655 min, Phlorizin; Std was a mixture of procyanidin A_1_, A_2_, and phlorizin standards).

Some phenolic compounds identified by UPLC‐Q/TOF‐MS were not quantified by HPLC, as they were present in low concentrations. Only the contents of major phenolic compounds (procyanidin A_1_, procyanidin A_2_, and phlorizin) were shown in Table 3 and Figure 2. A‐type procyanidin dimers were distinguished as procyanidin A_1_ and A_2_ in HPLC based on a comparison of their retention time with the standards. Procyanidin A_2_ was a dominant component in five polyphenol samples, ranging from 16.92 ± 0.23 (HZ) to 40.62 ± 0.38 (HY) mg/g DW. Procyanidin A_1_ contents were also highest in HY (24.92 ± 0.20 mg/g DW) and lowest in HZ (6.27 ± 0.13 mg/g DW). BTY and HY contained relatively higher contents of phlorizin, which were 2.25 ± 0.17 and 1.88 ± 0.02 mg/g DW, respectively. The contents of three phenolic compounds were significantly different among five samples (*p* < 0.05), with relatively rich amounts in HY. Although procyanidin A_1_ and A_2_ had been accurately identified in the litchi seeds in a previous study of Xu et al. (2010), their contents in different varieties had seldom been documented. Man et al. (2016) had measured the content of phlorizin in litchi seed polyphenol extract as 1.33 mg/g, near to the results in our study.

**TABLE 3:** Contents of procyanidin A_1_, A_2_, and phlorizin in BTY, BL, FZX, HY, and HZ.

Variety	Procyanidin A_1_ (mg/g DW)	Procyanidin A_2_ (mg/g DW)	Phlorizin (mg/g DW)
HY	24.92 ± 0.20^a^	40.62 ± 0.38^a^	1.88 ± 0.02^b^
FZX	14.22 ± 0.10^d^	31.01 ± 0.22^b^	0.61 ± 0.05^e^
BL	21.07 ± 0.60^b^	29.68 ± 0.58^c^	1.02 ± 0.00^d^
BTY	19.44 ± 0.18^c^	28.24 ± 0.25^d^	2.25 ± 0.17^a^
HZ	6.27 ± 0.13^e^	16.92 ± 0.23^e^	1.22 ± 0.04^c^

*Note:* Mean ± SD (*n* = 3) labeled with different letters across the five varieties differ significantly (*p* < 0.05).

### 3.4 Anti‐Inflammatory and Analgesic Potentials in RAW 264.7 Macrophages

#### 3.4.1 Cell Viability

**FIGURE 3:**
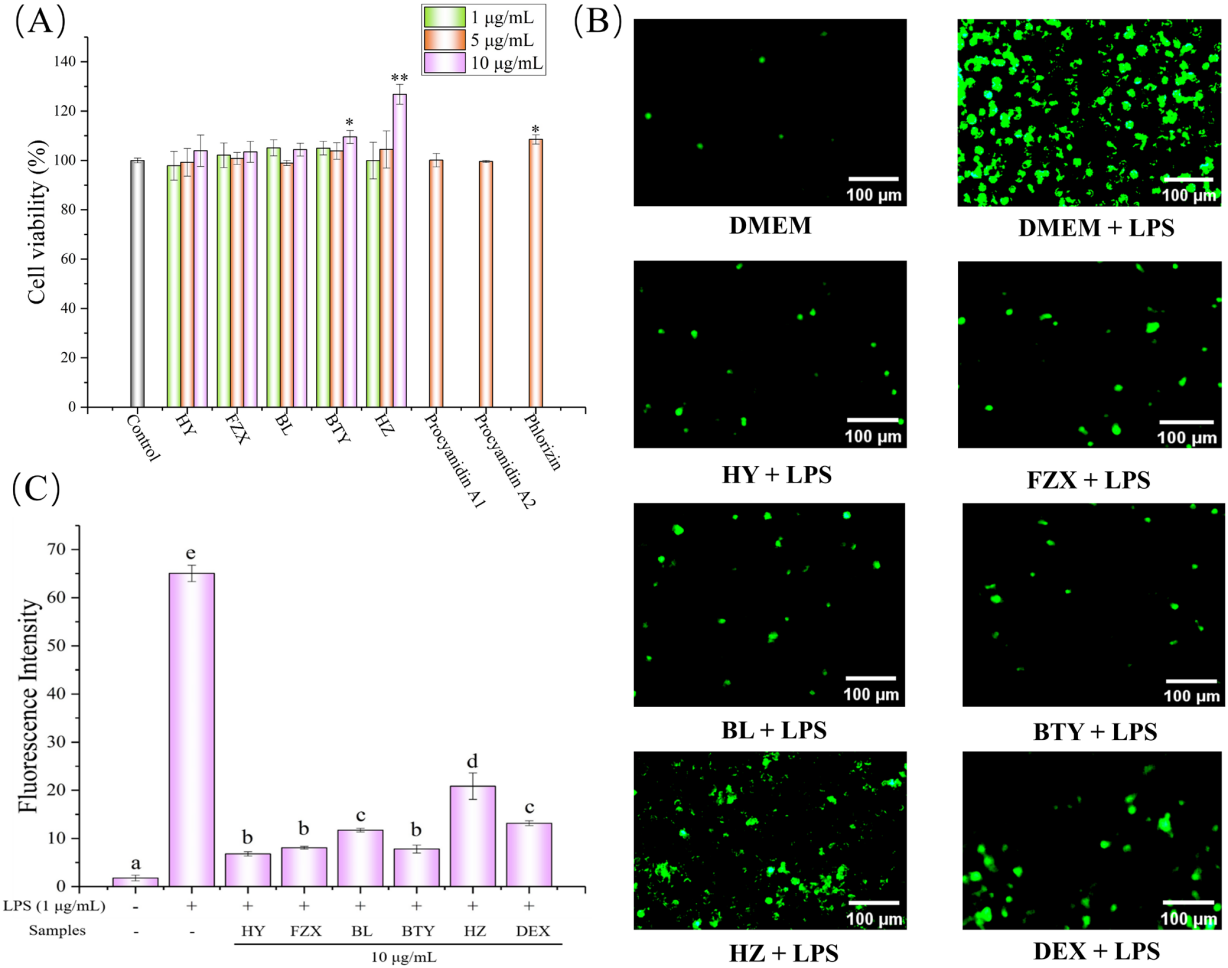
(A) Effect of BTY, BL, FZX, HY, HZ, and individual phenolic compounds on the viability of RAW 264.7 macrophages. Asterisks (*) denoted a statistically significant difference from the control (*p < 0.05, **p < 0.01; *n* = 3). (B) Representative fluorescence images (scale bar = 100 μm) and (C) fluorescence intensity of the intracellular ROS levels in RAW 264.7 macrophages. The presence of different letters above the bars indicated a statistically significant difference between the mean ± SD (*p* < 0.05, *n* = 3).

To assess the in vitro anti‐inflammatory and analgesic properties of BTY, BL, FZX, HY, HZ, and individual phenolic compounds, a cytotoxicity assay was first conducted on RAW 264.7 macrophages. CCK‐8 method was performed after 24 h of sample treatment. As depicted in Figure 3A, compared to the control group, 5 μg/mL of phlorizin, 10 μg/mL of BTY (*p* < 0.05), and 10 μg/mL of HZ (*p* < 0.01) significantly promoted the proliferation of the RAW 264.7 cells. In addition, all the groups did not exhibit a significantly inhibitory effect on the cell viability, with the survival rates above 95%. Therefore, no cytotoxic effect was observed in the RAW 264.7 macrophages following treatment with the samples at concentrations ranging from 1 to 10 μg/mL. Accordingly, subsequent experiments were conducted within the concentration range.

#### 3.4.2 ROS Production

ROS play a significant role in immune defenses. However, excessive ROS generation, acting as mediators, leads to oxidative stress and chronic inflammatory diseases. Inflammatory pain is closely linked to oxidative stress. Phenolic compounds in the litchi seeds such as procyanidin B_2_, epicatechin, and protocatechuic acid were reported to contribute to the antioxidant activity (Nagendra Prasad et al. 2009; Wang et al. 2011). The effect of BTY, BL, FZX, HY, and HZ (10 μg/mL) on LPS‐induced ROS production in the RAW 264.7 cells was investigated using a ROS‐sensitive fluorescence indicator DCFH‐DA. In viable cells, the DCFH‐DA was cleaved by esterase to form DCFH, which was subsequently oxidized by ROS into a highly fluorescent compound DCF. The results in Figure 3B,C depicted that the intracellular levels of ROS, indicated by the fluorescence intensity, were significantly up‐regulated after 24 h‐treatment with LPS (*p* < 0.05). In contrast, the LPS‐induced ROS production was significantly reduced with the pretreatment with 10 μg/mL of BTY, BL, FZX, HY, and HZ (*p* < 0.05). Notably, HZ exhibited significantly lower inhibitory ability, probably related to their significantly lower contents of procyanidin A_1_ and A_2_ (*p* < 0.05). Procyanidins can enhance cellular antioxidant defenses through direct ROS molecular scavenging and regulation of various signaling pathways, such as MAPK, NF‐κB, and Nrf2 (nuclear factor erythroid 2‐related factor 2). Furthermore, these pathways are regulated along with ROS reduction and inflammatory response inhibition (Yang et al. 2018).

#### 3.4.3 NO, IL‐6, TNF‐α, PGE_2_ Production and Molecular Contribution

**FIGURE 4:**
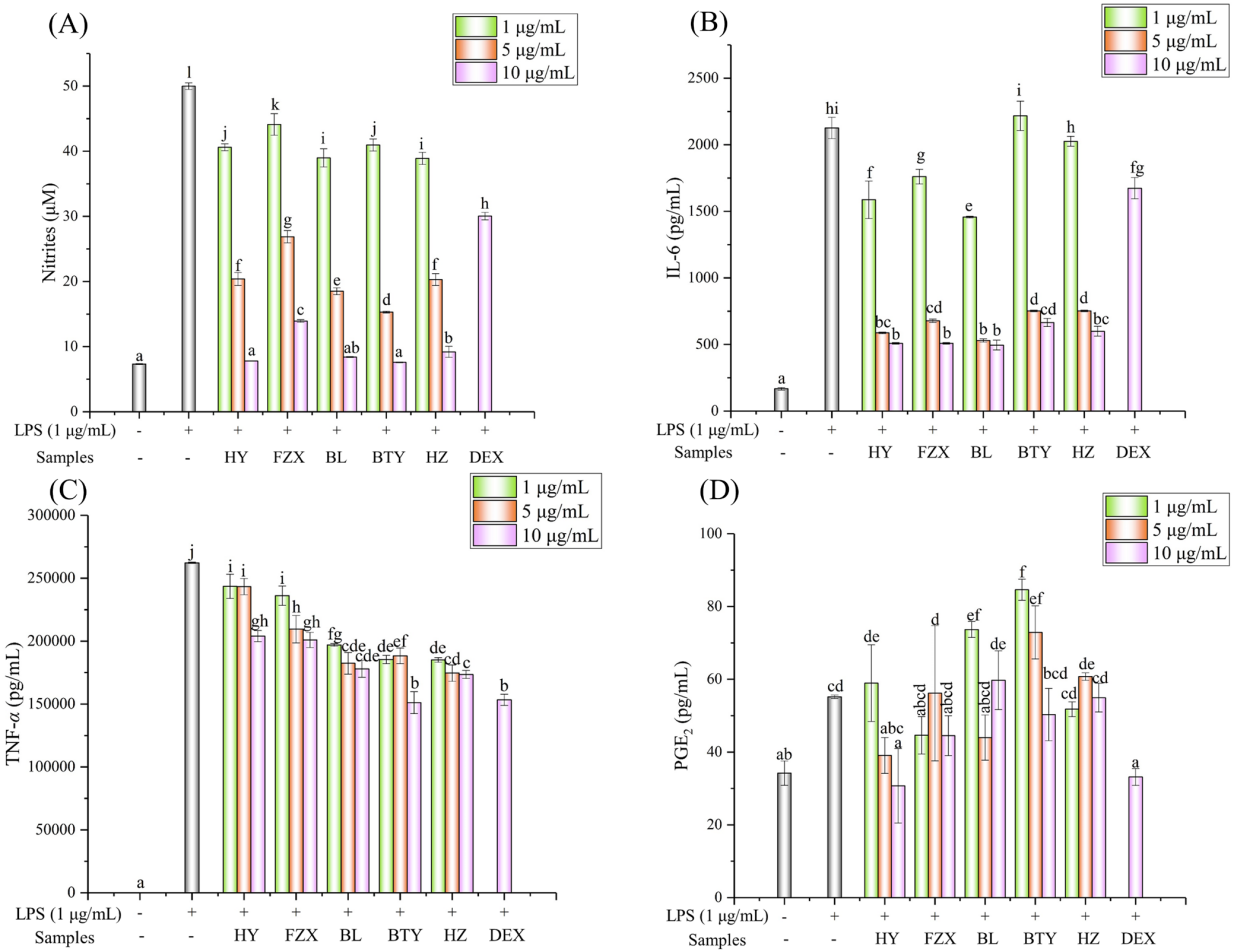
Effect of BTY, BL, FZX, HY, and HZ on LPS‐induced production of nitrites (A), IL‐6 (B), TNF‐*α* (C), and PGE_2_ (D). Mean values (*n* = 3) labeled with distinct letters in every index showed significant difference (*p* < 0.05).

LPS is a well‐known potent inducer of inflammation, stimulating macrophages to release various cytokines. It is commonly used to check anti‐inflammatory drug in the RAW 264.7 macrophages. As shown in Figure 4A, BTY, BL, FZX, HY, and HZ (1–10 μg/mL) significantly inhibited LPS‐induced nitrite production in a dose‐dependent manner (*p* < 0.05), which was equal to NO production. The NO levels in the groups with pretreatment of 10 μg/mL of HY, BL, and BTY were 7.80, 8.42, and 7.58 μM respectively, which were significantly lower than those in other varieties and even DEX groups at the same concentration (*p* < 0.05). The production of pro‐inflammatory cytokines such as IL‐6, TNF‐*α*, and PGE_2_ was significantly raised by LPS up to 12.55, 3943.29, and 1.61 folds respectively when compared to the control without LPS treatment (*p* < 0.05). Five to10 μg/mL of litchi seed polyphenols could remarkably inhibit the excessive production of IL‐6, especially for HY and BL, which were more effective than the positive drug DEX (Figure 4B, *p* < 0.05). One to 10 μg/mL of litchi seed polyphenols could significantly attenuate the excessive production of TNF‐*α* and the highest inhibition rate was detected above 40% for 10 μg/mL of BTY and DEX (Figure 4C, *p* < 0.05). In response to LPS stimulation, pro‐inflammatory cytokine PGE_2_ production was related with pathological pain. Obvious inhibition effect on PGE_2_ production was only found for 10 μg/mL of HY, which was comparable to DEX at the same concentration (Figure 4D, *p* < 0.05). Hence, it could be concluded that among five cultivars, HY exhibited relatively better ability to suppress LPS‐induced production of NO and pro‐inflammatory cytokines.

**FIGURE 5:**
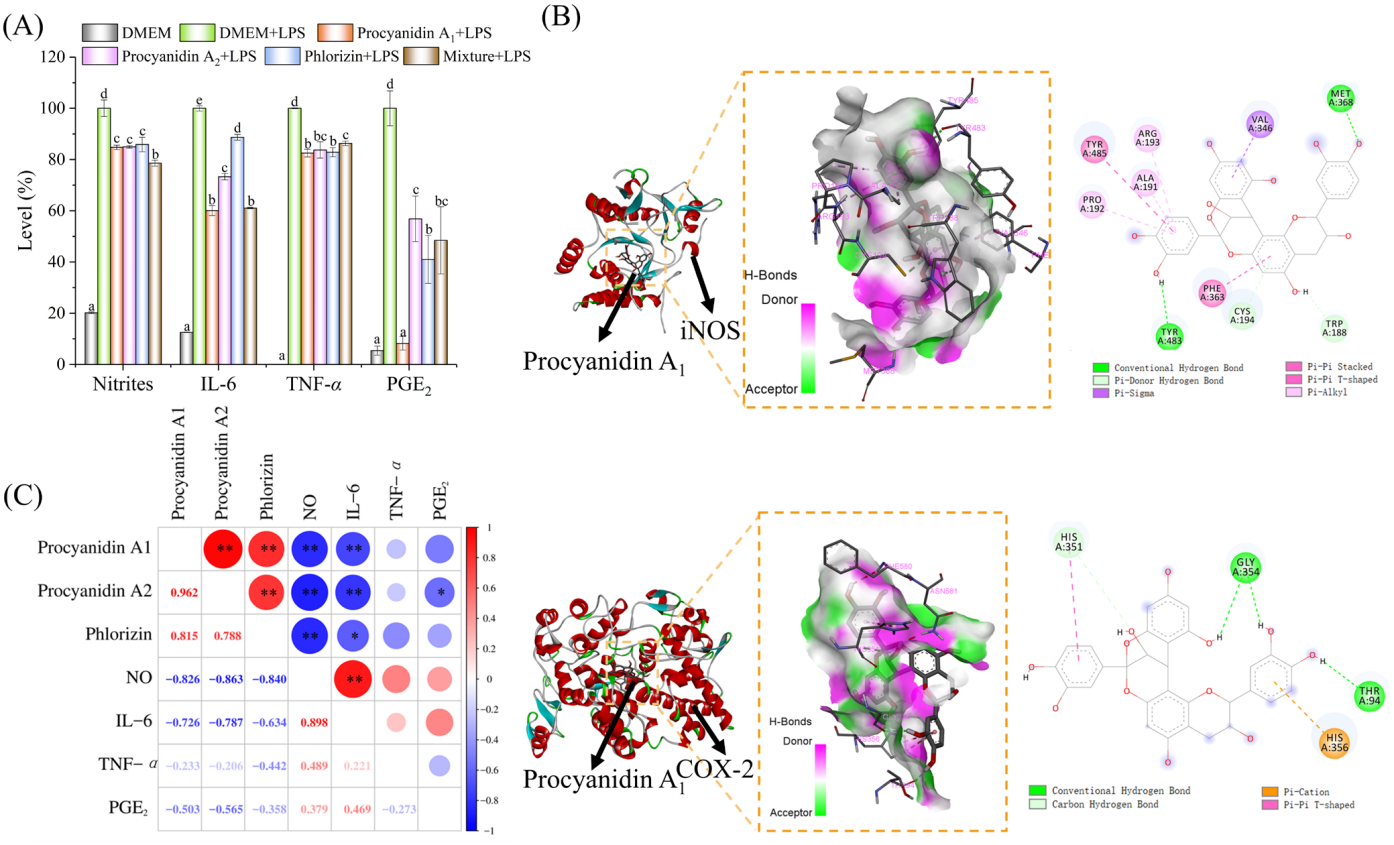
(A) Effect of procyanidin A_1_, procyanidin A_2_, phlorizin, and their mixture (quality ratio 20∶30∶2) on LPS‐induced production of nitrites, IL‐6, TNF‐*α*, and PGE_2_. Significant difference (*p* < 0.05) was denoted by different letters above the mean bars (*n* = 3). (B) Molecular docking between procyanidin A_1_ and iNOS, COX‐2. (C) Correlation coefficients between the concentration of procyanidin A_1_, procyanidin A_2_, phlorizin in the litchi seed polyphenols and production of NO, IL‐6, TNF‐*α*, PGE_2_. Statistical significance was determined using a 2‐tailed test, with correlations marked at significance levels of 0.01** and 0.05*.

Furthermore, the contribution of major phenolic compounds (procyanidin A_1_, procyanidin A_2_, and phlorizin) to the in vitro anti‐inflammatory and analgesic effect was also evaluated. As shown in Figure 5A, three phenolic compounds at a concentration of 5 μg/mL could significantly inhibit the production of NO, IL‐6, TNF‐*α*, and PGE_2_ (*p* < 0.05). As for the NO and TNF‐*α* inhibition ability, procyanidin A_1_, procyanidin A_2_, and phlorizin showed no obvious difference (*p* > 0.05). On the other hand, procyanidin A_1_ exhibited a significantly stronger inhibition effect on IL‐6 and PGE_2_ production (*p* < 0.05). To further investigate whether procyanidin A_1_, procyanidin A_2_, and phlorizin exhibited a better effect in a mixture, it was prepared with a quality ratio of 20∶30∶2 which was a moderate value among different litchi seeds, especially for HY and BTY with relatively better activity. As the results suggested, a better effect of the mixture was only detected in NO inhibition (*p* < 0.05). The mixture exhibited strong ability to inhibit the IL‐6 production without significant difference from procyanidin A_1_ (*p* > 0.05). However, the strong ability of procyanidin A_1_ to inhibit PGE_2_ production was significantly attenuated in the mixture (*p* < 0.05). Peptide hydrolysates and methyl jasmonate analogs had been previously considered as anti‐inflammatory agents in the litchi seeds, suppressing the production of NO and IL‐6 (Appeldoorn et al. 2009; Saisavoey et al. 2018). In this study, procyanidin A_1_ was demonstrated to be another anti‐inflammatory and analgesic agent in the litchi seeds. In the molecular simulation, the re‐docking procedure yielded a RMSD value of 1.426 and 1.809 for iNOS and COX‐2 respectively, validating the accuracy of the docking methodology used in this study. As a result, procyanidin A_1_ could tightly bind to iNOS and COX‐2 with binding energies of −8.9 and −8.4 kcal/mol, respectively. Figure 5B showed the interaction between the ligand and target proteins. Procyanidin A_1_ formed hydrogen bonds with iNOS and COX‐2 at specific amino acid sites including MET368, TYR483, GLY354, and THR94 (dark green discs). iNOS and COX‐2 act as key pathway enzymes to catalyze the synthesis of NO and PGE_2_, respectively. Through binding to the catalytic domains of iNOS and COX‐2, the ligand seemed to change the protein conformation and inhibit the catalytic reaction (Lescano et al. 2018). Thus, procyanidin A_1_ probably acted as a promising inhibitor. The mechanisms of regulating inflammation signaling pathways should be further investigated.

#### 3.4.4 Correlation Coefficients Between Phenolic Contents in Litchi Seed Polyphenols and in Vitro Activities

The correlations between the phenolic contents in BTY, BL, FZX, HY, HZ, and production of NO, IL‐6, TNF‐*α*, PGE_2_ were analyzed using the statistical method, and the results were shown in Figure 5C. The concentration of procyanidin A_1_, procyanidin A_2_, and phlorizin was highly negatively correlated to the NO production (*p* < 0.01, *r* = −0.826, −0.863, and −0.840, respectively). Strong negative correlations were also detected between the IL‐6 production and concentration of procyanidin A_1_ (*p* < 0.01, *r* = −0.726), procyanidin A_2_ (*p* < 0.01, *r* = −0.787), and phlorizin (*p* < 0.05, *r* = −0.634). This indicated the NO and IL‐6 inhibition process of litchi seed polyphenols was tightly associated with the contents of these three phenolic compounds. Only procyanidin A_2_ exhibited a significant inverse correlation with the PGE_2_ production (*p* < 0.05, *r* = −0.565). Meanwhile, none of the three compounds showed a significant correlation with the TNF‐*α* production (*p* > 0.05), which might be regulated by other phenolic compounds in the litchi seed polyphenols. Xie et al. (2023) discovered that the concentration of A‐type procyanidin trimers in litchi fruitlets was significantly correlated with the NO inhibition rates, and the correlation coefficient was 0.53, which was much lower than that of A‐type procyanidin dimers in our study.

### 3.5 Anti‐Inflammatory Activity of HY in the Xylene‐Induced Mouse Ear Edema and Analgesic Activity in the Acetic Acid‐Induced Mouse Abdominal Writhing Response

**FIGURE 6:**
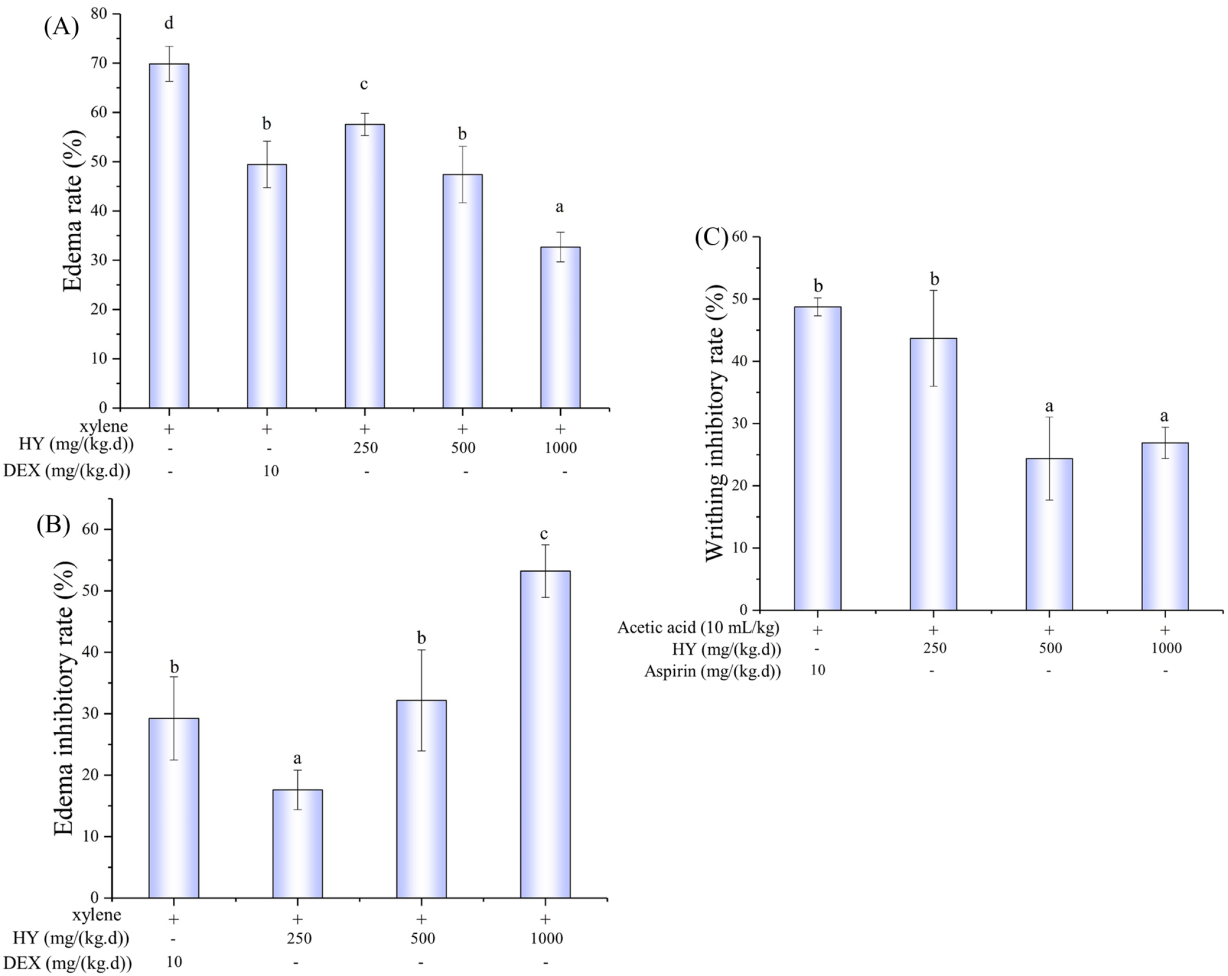
Edema rate (A) and edema inhibitory rate (B) in the xylene‐treated mouse ears and writhing inhibitory rate (C) in the acetic acid‐induced mouse abdominal writhing response. Results were presented as mean ± SD (*n* = 6). Bars bearing different superscript letters differed significantly (*p* < 0.05).

Considering the relatively higher phenolic contents and better in vitro activity of HY, it was further used to evaluate the anti‐inflammatory and analgesic potentials in vivo. As shown in Figure 6A, the ear edema of mice occurred (edema rate 69.86%) along with the topical administration of xylene on their right ears, in comparison with the left ears. The ear edema showed different degrees of relief with 250, 500, and 1000 mg/(kg·day) of HY intervention and the edema inhibitory rates were 17.60%, 32.17%, and 53.22%, respectively (Figure 6B). HY significantly attenuated xylene‐induced ear edema in a dose‐dependent manner (*p* < 0.05), and the effect of 500 mg/(kg·day) of HY was equal to the positive drug DEX [10 mg/(kg·day)]. Treatment with 0.60% acetic acid caused mouse abdominal writhing around 40 times within 10 min. Figure 6C suggested that the analgesic drug aspirin [10 mg/(kg·day)] inhibited the mouse abdominal writhing at a rate of 48.74%, comparable to HY of 250 mg/(kg·day). Interestingly, when the concentration of HY increased to 500 and 1000 mg/(kg·day), the inhibitory effect was significantly attenuated (*p* < 0.05), with the inhibitory rates of 24.37% and 26.89%, respectively. In peripheral tissues, abdominal injection of acetic acid induces generation of prostaglandins and the writhing is the consequence of sensitization of chemosensitive nociceptors by the prostaglandins. The contradictory dose responses in the regulation of ear edema and abdominal writhing could be explained by the difference in pharmacokinetics of constituents in HY, along with differentiated regulation on the inflammatory factors and prostaglandins (Abera and Hailu 2019). Accordingly, the higher dose of HY after metabolism had a greater effect as seen in the xylene‐induced ear edema, but the lower dose had a greater effect in the acetic acid‐induced abdominal writhing. Consequently, the litchi seed polyphenols exhibited anti‐inflammatory and analgesic potentials in vivo. Similarly aimed to support the traditional use of litchi seeds in folk medicine, Sindhu et al. (2021) had investigated the anti‐inflammatory and analgesic effects of the ethanolic crude extract of litchi seeds and screened the phytochemical constituents. Distinct from our study, the anti‐inflammatory and analgesic activities were assessed in Wistar albino rat models of carrageenan‐induced paw edema and a hot plate test, respectively. Orally administered at doses of 150, 300, and 600 mg/kg, the extract elicited significant inhibition and protective effects. Although phenols, tannins, flavonoids, saponins, steroids, and alkaloids were characterized in the extract, the relation between the phytochemical constituents and activities was indefinite. In contrast, our study provided a relatively detailed molecular basis to explain the traditional use of litchi seeds in alleviating inflammation and pain.

## 4 Conclusions

This study was dedicated to revealing the phenolic profiles in the litchi seeds of five different cultivars and their contributions to the litchi seeds' traditional application in anti‐inflammatory and analgesic aspects. The purified phenolic extract HY contained relatively higher proportions of total phenolic, flavonoid, and proanthocyanidin contents than those in other cultivars including BTY, BL, FZX, and HZ. Fifteen flavonoids, two phenolic acids, one coumarin, and two tannins were characterized. Procyanidin A_1_, procyanidin A_2_, and phlorizin were identified as major phenolic compounds. BTY, BL, FZX, HY, and HZ effectively inhibited the production of ROS, NO, IL‐6, TNF‐*α*, and PGE_2_ in LPS‐induced RAW 264.7 macrophages, which was tightly associated with the concentration of procyanidin A_1_, procyanidin A_2_, and phlorizin. Meanwhile, procyanidin A_1_ played a relatively significant role probably through strongly binding to iNOS and COX‐2, and their mixture with procyanidin A_2_ and phlorizin exhibited a better effect in the NO inhibition. Furthermore, HY was demonstrated to attenuate acute inflammation in the xylene‐induced mouse ear edema and show pain relief effect in the acetic acid‐induced mouse abdominal writhing response. Thus, litchi seed polyphenols possess potential applications as anti‐inflammatory and analgesic agents.

## Author Contributions

**Fanke Zeng:** conceptualization, data curation, formal analysis, funding acquisition, methodology, project administration, visualization, writing – original draft. **Xiaoyan Li:** validation. **Lei Fang:** funding acquisition. **Lei Pan:** writing – review and editing. **Shaodan Peng:** supervision, resources. **Wei Zhou:** funding acquisition, supervision, writing – review and editing. **Yuxiang Zhu:** data curation, investigation. **Yaping Dai:** resources. **Jihua Li:** funding acquisition, resources. **Ruyi Li:** software, writing – review and editing.

## Funding

This work was supported by the National Key R&D Program of China (grant number 2024YFD2100601); the Earmarked Fund for CARS‐30; the Central Public‐interest Scientific Institution Basal Research Fund for Chinese Academy of Tropical Agricultural Sciences (grant number 1630012025202, 1630012025215); the Innovative and Entrepreneurship Team Project for “Pilot Program” of Zhanjiang City (grant number 211207157080998); and the Tip‐top Scientific and Technical Innovative Youth Talents of Guangdong Special Support Program (grant number NYQN2024005).

## Conflicts of Interest

The authors declare no conflicts of interest.

## Data Availability

The data that support the findings of this study are available on request from the corresponding author. The data are not publicly available due to privacy or ethical restrictions.

## References

[fsn372200-bib-0001] Abera, B. , and A. E. Hailu . 2019. “Evaluation of Analgesic Activities of 80% Methanol Leaf Extract of *Solanum incanum* L. (Solanaceae) in Mice.” Journal of Drug Delivery and Therapeutics 9, no. 5: 9–14. 10.22270/jddt.v9i5.3235.

[fsn372200-bib-0002] Appeldoorn, M. M. , M. Sanders , J. P. Vincken , et al. 2009. “Efficient Isolation of Major Procyanidin A‐Type Dimers From Peanut Skins and B‐Type Dimers From Grape Seeds.” Food Chemistry 117, no. 4: 713–720. 10.1016/j.foodchem.2009.04.047.

[fsn372200-bib-0003] Chen, W. , D. Wang , L. S. Wang , et al. 2012. “Pharmacokinetics of Protocatechuic Acid in Mouse and Its Quantification in Human Plasma Using LC‐Tandem Mass Spectrometry.” Journal of Chromatography. B, Analytical Technologies in the Biomedical and Life Sciences 908: 39–44. 10.1016/j.jchromb.2012.09.032. 23122399 PMC3538353

[fsn372200-bib-0004] Cheng, Y. , C. Wu , Z. Liu , P. Song , B. Xu , and Z. Chao . 2023. “Evaluation and Optimization of Quality Based on the Physicochemical Characteristics and Metabolites Changes of Qingpi During Storage.” Foods 12: 463. 10.3390/foods12030463. 36765992 PMC9914837

[fsn372200-bib-0005] Chukwuma, C. I. , G. O. Izu , M. S. Chukwuma , M. S. Samson , T. J. Makhafola , and O. L. Erukainure . 2021. “A Review on the Medicinal Potential, Toxicology, and Phytochemistry of Litchi Fruit Peel and Seed.” Journal of Food Biochemistry 45, no. 12: e13997. 10.1111/jfbc.13997. 34750843

[fsn372200-bib-0006] da Silva, A. P. G. , W. G. Sganzerla , O. D. John , and R. Marchiosi . 2023. “A Comprehensive Review of the Classification, Sources, Biosynthesis, and Biological Properties of Hydroxybenzoic and Hydroxycinnamic Acids.” Phytochemistry Reviews 24, no. 2: 1061–1090. 10.1007/s11101-023-09891-y.

[fsn372200-bib-0007] Deng, Q. , W. Chen , B. Deng , et al. 2024. “Based on Network Pharmacology, Molecular Docking and Experimental Verification to Reveal the Mechanism of *Andrographis paniculata* Against Solar Dermatitis.” Phytomedicine 135: 156025. 10.1016/j.phymed.2024.156025. 39326136

[fsn372200-bib-0008] El‐Zahar, H. , E. T. Menze , H. Handoussa , et al. 2022. “UPLC‐PDA‐MS/MS Profiling and Healing Activity of Polyphenol‐Rich Fraction of *Alhagi maurorum* Against Oral Ulcer in Rats.” Plants 11, no. 3: 455. 10.3390/plants11030455. 35161436 PMC8838639

[fsn372200-bib-0009] Giusti, F. , G. Caprioli , M. Ricciutelli , S. Vittori , and G. Sagratini . 2017. “Determination of Fourteen Polyphenols in Pulses by High Performance Liquid Chromatography‐Diode Array Detection (HPLC‐DAD) and Correlation Study With Antioxidant Activity and Colour.” Food Chemistry 221: 689–697. 10.1016/j.foodchem.2016.11.118. 27979260

[fsn372200-bib-0010] Hilt, P. , A. Schieber , C. Yildirim , et al. 2003. “Detection of Phloridzin in Strawberries ( *Fragaria x ananassa* Duch.) by HPLC−PDA−MS/MS and NMR Spectroscopy.” Journal of Agricultural and Food Chemistry 51, no. 10: 2896–2899. 10.1021/jf021115k. 12720368

[fsn372200-bib-0011] Hossain, M. B. , D. K. Rai , N. P. Brunton , A. B. Martin‐Diana , and C. Barry‐Ryan . 2010. “Characterization of Phenolic Composition in Lamiaceae Spices by LC‐ESI‐MS/MS.” Journal of Agricultural and Food Chemistry 58, no. 19: 10576–10581. 10.1021/jf102042g. 20825192

[fsn372200-bib-0012] Ilari, S. , S. Proietti , P. Russo , et al. 2022. “A Systematic Review and Meta‐Analysis on the Role of Nutraceuticals in the Management of Neuropathic Pain in In Vivo Studies.” Antioxidants 11, no. 12: 2361. 10.3390/antiox11122361. 36552569 PMC9774415

[fsn372200-bib-0013] Jiang, N. , H. Zhu , W. Liu , C. Fan , F. Jin , and X. Xiang . 2021. “Metabolite Differences of Polyphenols in Different Litchi Cultivars ( *Litchi chinensis* Sonn.) Based on Extensive Targeted Metabonomics.” Molecules 26, no. 4: 1181. 10.3390/molecules26041181. 33672099 PMC7926386

[fsn372200-bib-0014] Kavya, M. K. , B. S. Suresha , T. Balasubramanian , and K. H. A. Devi . 2025. “A Review on *Litchi chinensis* .” International Journal of Pharmacognosy 12, no. 6: 471–477. 10.13040/ijpsr.0975-8232.Ijp.12(6).471-77.

[fsn372200-bib-0015] Kessy, H. N. , Z. Hu , L. Zhao , and M. Zhou . 2016. “Effect of Steam Blanching and Drying on Phenolic Compounds of Litchi Pericarp.” Molecules 21, no. 6: 729. 10.3390/molecules21060729. 27271581 PMC6273031

[fsn372200-bib-0016] Laily, N. , R. W. Kusumaningtyas , I. Sukarti , and M. R. D. K. Rini . 2015. “The Potency of Guava *Psidium guajava* (L.) Leaves as a Functional Immunostimulatory Ingredient.” Procedia Chemistry 14: 301–307. 10.1016/j.proche.2015.03.042.

[fsn372200-bib-0017] Lescano, C. H. , F. Freitas de Lima , C. B. Mendes‐Silvério , et al. 2018. “Effect of Polyphenols From *Campomanesia adamantium* on Platelet Aggregation and Inhibition of Cyclooxygenases: Molecular Docking and In Vitro Analysis.” Frontiers in Pharmacology 9: 617. 10.3389/fphar.2018.00617. 29946259 PMC6005896

[fsn372200-bib-0018] Li, C. , X. Qiu , X. Hou , et al. 2025. “Polymerization of Proanthocyanidins Under the Catalysis of miR397a‐Regulated Laccases in *Salvia miltiorrhiza* and *Populus trichocarpa* .” Nature Communications 16, no. 1: 1513. 10.1038/s41467-025-56864-0. PMC11811200 39929881

[fsn372200-bib-0019] Maisto, M. , V. Piccolo , E. Novellino , et al. 2022. “Optimization of Phlorizin Extraction From Annurca Apple Tree Leaves Using Response Surface Methodology.” Antioxidants 11, no. 10: 1933. 10.3390/antiox11101933. 36290654 PMC9598179

[fsn372200-bib-0020] Man, S. , J. Ma , C. Wang , Y. Li , W. Gao , and F. Lu . 2016. “Chemical Composition and Hypoglycaemic Effect of Polyphenol Extracts From *Litchi chinensis* Seeds.” Journal of Functional Foods 22: 313–324. 10.1016/j.jff.2016.01.032.

[fsn372200-bib-0021] Mitra, S. , A. M. Tareq , R. Das , et al. 2022. “Polyphenols: A First Evidence in the Synergism and Bioactivities.” Food Reviews International 39, no. 7: 4419–4441. 10.1080/87559129.2022.2026376.

[fsn372200-bib-0022] M'rabet, Y. , N. Rokbeni , S. Cluzet , et al. 2017. “Profiling of Phenolic Compounds and Antioxidant Activity of *Melia azedarach* L. Leaves and Fruits at Two Stages of Maturity.” Industrial Crops and Products 107: 232–243. 10.1016/j.indcrop.2017.05.048.

[fsn372200-bib-0023] Mukinda, J. T. , and P. F. Eagles . 2010. “Acute and Sub‐Chronic Oral Toxicity Profiles of the Aqueous Extract of *Polygala fruticosa* in Female Mice and Rats.” Journal of Ethnopharmacology 128, no. 1: 236–240. 10.1016/j.jep.2010.01.022. 20079821

[fsn372200-bib-0024] Nagendra Prasad, K. , B. Yang , S. Yang , et al. 2009. “Identification of Phenolic Compounds and Appraisal of Antioxidant and Antityrosinase Activities From Litchi (*Litchi sinensis* Sonn.) Seeds.” Food Chemistry 116, no. 1: 1–7. 10.1016/j.foodchem.2009.01.079.

[fsn372200-bib-0025] Oryza Oil & Fat Chemical Co., Ltd . 2019. Litchi Seed Extract. Oryza Oil & Fat Chemical Co., Ltd.

[fsn372200-bib-0026] Ren, S. , D. Xu , Z. Pan , Y. Gao , Z. Jiang , and Q. Gao . 2011. “Two Flavanone Compounds From Litchi ( *Litchi chinensis* Sonn.) Seeds, One Previously Unreported, and Appraisal of Their *α*‐Glucosidase Inhibitory Activities.” Food Chemistry 127, no. 4: 1760–1763. 10.1016/j.foodchem.2011.02.054.

[fsn372200-bib-0027] Saisavoey, T. , P. Sangtanoo , O. Reamtong , and A. Karnchanatat . 2018. “Anti‐Inflammatory Effects of Lychee ( *Litchi chinensis* Sonn.) Seed Peptide Hydrolysate on RAW 264.7 Macrophage Cells.” Food Biotechnology 32, no. 2: 79–94. 10.1080/08905436.2018.1443821.

[fsn372200-bib-0028] Shoaib, T. H. , N. Abdelmoniem , R. M. Mukhtar , et al. 2023. “Molecular Docking and Molecular Dynamics Studies Reveal the Anticancer Potential of Medicinal‐Plant‐Derived Lignans as MDM2‐P53 Interaction Inhibitors.” Molecules 28: 6665. 10.3390/molecules28186665. 37764441 PMC10536213

[fsn372200-bib-0029] Sindhu, K. , P. Bipindra , K. Sistu , G. Santosh , and G. Ashish . 2021. “Anti‐Inflammatory, Analgesic, and Acute Toxicity Evaluation of *Litchi chinensis* Seed Extract in Albino Rat.” Natural Resources for Human Health 1, no. 1: 30–35. 10.53365/nrfhh/141230.

[fsn372200-bib-0030] Su, D. , R. Zhang , F. Hou , et al. 2014. “Comparison of the Free and Bound Phenolic Profiles and Cellular Antioxidant Activities of Litchi Pulp Extracts From Different Solvents.” BMC Complementary and Alternative Medicine 14: 9. 10.1186/1472-6882-14-9. 24405977 PMC3893551

[fsn372200-bib-0031] Wang, H. , L. Chen , B. Yang , et al. 2023. “Structures, Sources, Identification/Quantification Methods, Health Benefits, Bioaccessibility, and Products of Isorhamnetin Glycosides as Phytonutrients.” Nutrients 15: 1947. 10.3390/nu15081947. 37111165 PMC10143801

[fsn372200-bib-0032] Wang, L. , G. Lou , Z. Ma , and X. Liu . 2011. “Chemical Constituents With Antioxidant Activities From Litchi ( *Litchi chinensis* Sonn.) Seeds.” Food Chemistry 126, no. 3: 1081–1087. 10.1016/j.foodchem.2010.11.133.

[fsn372200-bib-0033] Weymarn, L. B. V. , and S. E. Murphy . 2001. “Coumarin Metabolism by Rat Esophageal Microsomes and Cytochrome P450 2A3.” Chemical Research in Toxicology 14, no. 10: 1386–1392. 10.1021/tx010065v. 11599930

[fsn372200-bib-0034] Xiang, J. Y. , Y. Y. Chi , J. X. Han , et al. 2022. “ *Litchi chinensis* Seed Prevents Obesity and Modulates the Gut Microbiota and Mycobiota Compositions in High‐Fat Diet‐Induced Obese Zebrafish.” Food & Function 13, no. 5: 2832–2845. 10.1039/d1fo03991a. 35179169

[fsn372200-bib-0035] Xie, C. , K. Wang , X. Liu , G. Liu , Z. Hu , and L. Zhao . 2023. “Characterization and Bioactivity of A‐Type Procyanidins From Litchi Fruitlets at Different Degrees of Development.” Food Chemistry 405, no. Pt A: 134855. 10.1016/j.foodchem.2022.134855. 36368102

[fsn372200-bib-0036] Xu, X. , H. Xie , Y. Wang , and X. Wei . 2010. “A‐Type Proanthocyanidins From Lychee Seeds and Their Antioxidant and Antiviral Activities.” Journal of Agricultural and Food Chemistry 58, no. 22: 11667–11672. 10.1021/jf1033202. 20964424

[fsn372200-bib-0037] Yang, B. , H. Wang , N. Prasad , Y. Pan , and Y. Jiang . 2011. “Use of Litchi (*Litchi sinensis* Sonn.) Seeds in Health.” In Nuts and Seeds in Health and Disease Prevention, 699–703. Elsevier.

[fsn372200-bib-0038] Yang, L. , D. Xian , X. Xiong , R. Lai , J. Song , and J. Zhong . 2018. “Proanthocyanidins Against Oxidative Stress: From Molecular Mechanisms to Clinical Applications.” BioMed Research International 2018: 8584136. 10.1155/2018/8584136. 29750172 PMC5884402

[fsn372200-bib-0039] Yang, X. , S. Liu , Y. Liu , et al. 2025. “Total Flavonoids of Litchi Seed Inhibit Breast Cancer Metastasis by Regulating the PI3K/AKT/mTOR and MAPKs Signaling Pathways.” Pharmaceutical Biology 63, no. 1: 229–249. 10.1080/13880209.2025.2488135. 40231974 PMC12001861

[fsn372200-bib-0040] Yang, Z. , L. Zhang , Y. H. Wu , D. P. Li , and W. Li . 2022. “Evaluation of Chemical Constituents of Litchi Pericarp Extracts and Its Antioxidant Activity in Mice.” Foods 11: 3837. 10.3390/foods11233837. 36496645 PMC9740626

[fsn372200-bib-0041] Yao, Y. , T. Liu , L. Yin , S. Man , S. Ye , and L. Ma . 2021. “Polyphenol‐Rich Extract From *Litchi chinensis* Seeds Alleviates Hypertension‐Induced Renal Damage in Rats.” Journal of Agricultural and Food Chemistry 69, no. 7: 2138–2148. 10.1021/acs.jafc.0c07046. 33470120

[fsn372200-bib-0042] Yu, H. H. , Y. Lin , R. Zeng , et al. 2019. “Analgesic and Anti‐Inflammatory Effects and Molecular Mechanisms of *Kadsura heteroclita* Stems, an Anti‐Arthritic Chinese Tujia Ethnomedicinal Herb.” Journal of Ethnopharmacology 238: 111902. 10.1016/j.jep.2019.111902. 31018145

[fsn372200-bib-0043] Zhang, H. , and R. Tsao . 2016. “Dietary Polyphenols, Oxidative Stress and Antioxidant and Anti‐Inflammatory Effects.” Current Opinion in Food Science 8: 33–42. 10.1016/j.cofs.2016.02.002.

[fsn372200-bib-0044] Zhang, R. , Q. Zeng , Y. Deng , et al. 2013. “Phenolic Profiles and Antioxidant Activity of Litchi Pulp of Different Cultivars Cultivated in Southern China.” Food Chemistry 136, no. 3–4: 1169–1176. 10.1016/j.foodchem.2012.09.085. 23194511

[fsn372200-bib-0045] Zhao, J. , A. Maitituersun , C. Li , et al. 2018. “Evaluation on Analgesic and Anti‐Inflammatory Activities of Total Flavonoids From *Juniperus sabina* .” Evidence‐Based Complementary and Alternative Medicine 2018: 7965306. 10.1155/2018/7965306. 30069226 PMC6057303

[fsn372200-bib-0046] Zheng, B. , Y. Yuan , J. Xiang , et al. 2022. “Green Extraction of Phenolic Compounds From Foxtail Millet Bran by Ultrasonic‐Assisted Deep Eutectic Solvent Extraction: Optimization, Comparison and Bioactivities.” LWT ‐ Food Science and Technology 154: 112740. 10.1016/j.lwt.2021.112740.

